# Assessment of Visual Quality in Eyes with Forme Fruste Keratoconus and Mild and Moderate Keratoconus Based on Optical Quality Analysis System II Parameters

**DOI:** 10.1155/2020/7505016

**Published:** 2020-02-29

**Authors:** Zhonghao Ren, Liyan Xu, Qi Fan, Kaili Yang, Shengwei Ren, Dongqing Zhao

**Affiliations:** ^1^Henan University People's Hospital, Henan Provincial People's Hospital, Henan Eye Hospital, Henan Eye Institute, Zhengzhou 450003, China; ^2^Henan Provincial People's Hospital, Henan Eye Hospital, Henan Eye Institute, People's Hospital of Zhengzhou University, Henan University People's Hospital, Zhengzhou 450003, China

## Abstract

**Purpose:**

The study aimed to evaluate the visual quality of forme fruste keratoconus (FFK) and mild and moderate keratoconus by using an optical quality analysis system II (OQAS-II) and to explore the correlation between optical quality parameters and the disease progression.

**Methods:**

Twenty-one normal eyes, twenty-one FFK eyes, twenty-one mild keratoconus eyes, and twenty-one moderate keratoconus eyes were included in this prospective study. The optical quality parameters, such as object scatter index (OSI), modulation transfer function cutoff (MTF cutoff), strehl ratio (SR), and OQAS-II values at contrasts of 100% (OV-100), 20% (OV-20), and 9% (OV-9), were measured by OQAS-II. The repeatability of these parameters was analyzed by intraclass correlation coefficient (ICC), repeatability coefficient (RC), and coefficient of variation (CV_w_). Correlations between optical quality parameters and mean central keratometry readings (*K*_mean_) were evaluated. The sensitivity and specificity of the parameters were analyzed using the receiver operating characteristic (ROC).

**Results:**

All the optical quality parameters among four groups showed good repeatability (all ICC≥0.75). The MTF cutoff, SR, OV-100, OV-20, OV-9 in FFK, mild and moderate keratoconus eyes were significantly lower than those in the normal group (all *P* < 0.05). The ROC analyses of the MTF cutoff, SR, OV-100, OV-20, and OV-9 showed significant area under the curve (AUC) in discriminating FFK form normal, mild keratoconus from FFK, and moderate keratoconus from mild keratoconus (all *P* < 0.05). The ROC analyses of the MTF cutoff, SR, OV-100, OV-20, and OV-9 showed significant area under the curve (AUC) in discriminating FFK form normal, mild keratoconus from FFK, and moderate keratoconus from mild keratoconus (all *P* < 0.05). The ROC analyses of the MTF cutoff, SR, OV-100, OV-20, and OV-9 showed significant area under the curve (AUC) in discriminating FFK form normal, mild keratoconus from FFK, and moderate keratoconus from mild keratoconus (all *P* < 0.05). The ROC analyses of the MTF cutoff, SR, OV-100, OV-20, and OV-9 showed significant area under the curve (AUC) in discriminating FFK form normal, mild keratoconus from FFK, and moderate keratoconus from mild keratoconus (all *P* < 0.05). The ROC analyses of the MTF cutoff, SR, OV-100, OV-20, and OV-9 showed significant area under the curve (AUC) in discriminating FFK form normal, mild keratoconus from FFK, and moderate keratoconus from mild keratoconus (all *K*_mean_) were evaluated. The sensitivity and specificity of the parameters were analyzed using the receiver operating characteristic (ROC). *r* = −0.710, *P* < 0.05). The ROC analyses of the MTF cutoff, SR, OV-100, OV-20, and OV-9 showed significant area under the curve (AUC) in discriminating FFK form normal, mild keratoconus from FFK, and moderate keratoconus from mild keratoconus (all

**Conclusion:**

The repeatability of OQAS-II is good in measuring visual quality of normal as well as FFK, mild, and moderate keratoconus. The visual quality of the FFK, mild, and moderate keratoconus is worse than that in normal eyes. The OQAS-II has the potential value in screening FFK from normal eyes and might be a useful tool for evaluating the progression of keratoconus.

## 1. Introduction

Keratoconus is a degenerative disorder characterized by corneal thinning and conical-shaped protrusion of the cornea [1]. It usually happens in adolescence and progresses until the third or fourth decade of life [2]. The progressive corneal protrusion and thinning would induce irregular astigmatism, leading to the impairment of visual function [3]. The individuals with mild keratoconus usually have their vision corrected with contact lenses or spectacles [4]. Nevertheless, the majority of keratoconus patients still have ocular discomfort and poor vision quality [5].

Several studies have found that the keratoconus patients have bad visual quality by questionnaire investigations [6,7]. Furthermore, it has been identified that the mild and moderate keratoconus had lower grades than normal people according to the National Eye Institute Visual Function Questionnaire-25 [8]. In addition, several studies have shown that both ocular and corneal aberrations are significantly higher in keratoconic eyes than those in normal eyes [9,10]. With the continuous development of digital image and computer processing, some new quantitative evaluation technology of the visual quality appeared. The optical quality analysis system (OQAS-II) is a double-pass (DP) system that could provide an objective clinical evaluation of the visual quality and has been successfully used to objectively classify the maturity of cataracts [11]. As far as we know, there are few studies aiming to evaluate the visual quality in keratoconus using OQAS-II. Ye et al. [12] found that the modulation transfer function cutoff (MTF cutoff), Strehl ratio (SR), OQAS values at contrasts of 100% (OV-100), OQAS values at contrasts of 20% (OV-20), and OQAS values at contrasts of 9% (OV-9) had significant differences between the normal and forme fruste keratoconus (FFK) patients. Leonard et al. [13] reported that the OSI was increased in keratoconic eyes and could be considered as a clinically significant parameter to stage keratoconus by directly evaluating visual quality. However, to the best of our knowledge, comparisons of all the parameters among FFK, mild keratoconus, moderate keratoconus patients, and normal have not yet been reported.

Therefore, the present study aimed to investigate the measurement variability of the OQAS-II and evaluate the visual quality of FFK, mild, and moderate keratoconus by OQAS-II. Additionally, we investigated the diagnostic ability of OQAS-II in screening FFK from normal eyes and explored the correlation between optical quality parameters and the disease progression.

## 2. Materials and Methods

### 2.1. Study Subjects

This study was conducted according to the Declaration of Helsinki guidelines, and all procedures involving human subjects were approved by the Institutional Review Board of Henan Eye Hospital. Written informed consents were obtained from all subjects.

Twenty-one FFK patients, 21 mild keratoconus patients and 21 moderate keratoconus patients were recruited in the refractive surgery center of Henan Eye Hospital from March 2018 to March 2019. Twenty-one simple refractive errors patients with matched age and gender were enrolled as the normal group. The FFK group consisted of 21 topographically normal eyes of patients with KC in the other eye [14]. The diagnosis of keratoconus was based on clinical examinations and the presence of characteristic corneal topographic features [15], in which the patients were presented with the eccentric steepening keratometry and anterior and the posterior elevation patterns such as I-S asymmetry, as well as at least one of the clinical diagnostic signs such as Fleischer ring, Vogt's striae, and corneal thinning by means of silt-lamp biomicroscopy. According to the Amsler-Krumeich scales (Supplemental [Supplementary-material supplementary-material-1]), stage 1 of the Amsler-Krumeich scales (AK1) and stage 2 of the Amsler-Krumeich scales (AK2) were defined as the mild and moderate keratoconus, respectively [15]. In patients with FFK, the fellow eye with keratoconus was excluded from the mild and moderate group. Eyes with previous ocular surgery, corneal haze, scar, cataract, vitreous opacity or aqueous humor opacity, rigid contact lens wears within 4 weeks, soft contact lens wears within 2 weeks, and severe keratoconus were excluded.

### 2.2. Clinical Examination

For each participant, a complete eye examination was performed, including best-corrected visual acuity (BCVA, logMAR), manifest refraction, silt-lamp and fundus examination, Visante Omni anterior segment OCT (Carl Zeiss Jena GmBH, Germany), corneal thickness in the thinnest point, intraocular pressure, and the axial length.

OQAS-II (Visiometrics, Spain) was used to measure the optical quality parameters of all subjects. The light source of the system is a 780 nm laser diode which is fully filtered and collimated. The point light source is imaged on the retina. After retina reflection, light passes twice through ocular media. Then, the HD Analyzer analyses the size and the shape of the reflected light spot. The measurement was performed in a darkroom to avoid the effects of spherical aberration and astigmatism. The subjects adapt to the dark environment for 10 minutes to acquire the largest pupil in the natural state after wearing the corrective lenses. The subjects were instructed to remain stationary after being positioned on the chin rest of the instrument. It was made sure that the subjects continued to fixate on the target and blink prior to each measurement to maintain a good tear film during the examination. The pupil diameter was set at 4 mm, and the spherical refraction errors were corrected by an incorporated optometer in the DP system (+5D∼−8D). For subjects with more than 0.5 D cylindrical refraction errors, the astigmatism was corrected with an external cylindrical lens [16]. All subjects underwent 3 consecutive tests and were measured by the same investigator with 5 minutes interval. The average value was used in the current analysis.

OQAS-II can provide six optical quality parameters, including OSI, MTF cut off, SR, OV-100, OV-20, and OV-9. The OSI quantifies intraocular scattered light and is defined as the ratio of the light of peripherally annular area versus that of the central peak in the acquired system. Small value of OSI is usually linked to eye with low scattering. The MTF cutoff is set as the cutoff point of the MTF curve on the *x*-axis and is calculated from the point spread function directly. The cutoff value represents the highest spatial frequency at which the MTF reaches the lowest contrast of 1%. The SR is defined as the ratio of the area under the MTF curve of the measured eye to that of the ideal aberration-free eye. The three OVs are normalized values of three spatial frequencies that correspond to the MTF values for three contrast conditions: 100 percent (OV-100), 20 percent (OV-20), and 9 percent (OV-9).

### 2.3. Statistical Analysis

All statistical analyses were performed by the SPSS 22.0 package (SPSS Inc., Chicago, IL, USA). Quantitative variables were expressed with mean ± standard deviation (SD) and analyzed by one-way analysis of variance with least significance difference (LSD-t) corrections. The qualitative variables were expressed with percentage and analyzed by Pearson's chi squared test. The measurement repeatability of optical quality parameters was assessed through three indicators, which include intraclass correlation coefficient (ICC), repeatability coefficient (RC), and coefficient of variation (CV_w_). For calculation, RC was defined as 2.77 times the intravisit within-subject SD (*S*_w_). The calculation of S_w_ was described as the square root of the within-subject mean square of error (the unbiased estimator of the component of variance because of random error) in a one-way random effects model [17]. CV_w_ was defined as 100 times *S*_w_ and then divided by the overall mean. The relationship between variables was analyzed using the bivariate correlation model and the Pearson correlation coefficient. For those parameters with statistically significant difference, receiver operating characteristic (ROC) analyses were performed to demonstrate the accuracy of the parameters in distinguishing FFK from normal eyes, AK1 from FFK, and AK2 from AK1. *P* values less than 0.05 were considered statistically significant.

## 3. Results

### 3.1. Demographic Data of Subjects

The demographic data of the four groups are shown in Table 1. The results showed no significant difference in gender, age, and the axial length among the 4 groups (all *P* > 0.05). The spherical equivalent in the AK2 group was significantly higher than that in the normal group (*P* < 0.05), while the spherical equivalent in AK1 group showed no significant difference with the FFK group as well as the normal group (all *P* < 0.05). The astigmatism, steep keratometry (*K*_s_), flat keratometry (*K*_f_), mean central keratometry readings (*K*_mean_), and BCVA (logMAR) in the AK1 group and AK2 group were significantly higher than those in the normal group (all *P* < 0.05), while the corneal thickness in the thinnest point and intraocular pressure in the AK1 group and AK2 group were significantly lower than those in the normal group (*P* < 0.05).

### 3.2. Repeatability of Optical Quality Parameters

Table 2 summarized the repeatability values of the OQAS-II parameters. In the normal group, 5 of 6 parameters (83.33%) showed excellent repeatability (ICC ≥ 0.90) and 1 parameter (16.67%) showed good repeatability (0.90 > ICC ≥ 0.75). Similarly, 5 of 6 parameters (83.33%) showed excellent repeatability (ICC ≥ 0.90) and 1 parameter (16.67%) showed good repeatability (0.90 > ICC ≥ 0.75) in FFK. All the parameters (100%) showed excellent repeatability (ICC ≥ 0.90) in AK1, while only 1 parameter (16.67%) showed excellent repeatability (ICC ≥ 0.90) and 5 parameters (83.33%) showed good repeatability (0.90 > ICC ≥ 0.75) in AK2.

### 3.3. Comparison of Optical Quality Parameters

The comparison of optical quality parameters among the four groups is shown in Table 3. The MTF cutoff, SR, OV-100, OV-20, OV-9 in FFK, AK1, and AK2 were significantly lower than those in the normal group (all *P* < 0.05). Similarly, the MTF cutoff, SR, OV-100, OV-20, and OV-9 values in the AK1 group and AK2 group were significantly lower than those in the FFK group (all *P* < 0.05). The OSI values in the AK1 group and AK2 group were significantly higher than those in the FFK group and normal group (*P* < 0.05), while the OSI values showed no significant difference between the FFK group and normal group.

### 3.4. The Relationship between the Optical Quality Parameters and *K*_mean_

**Figure 1:**
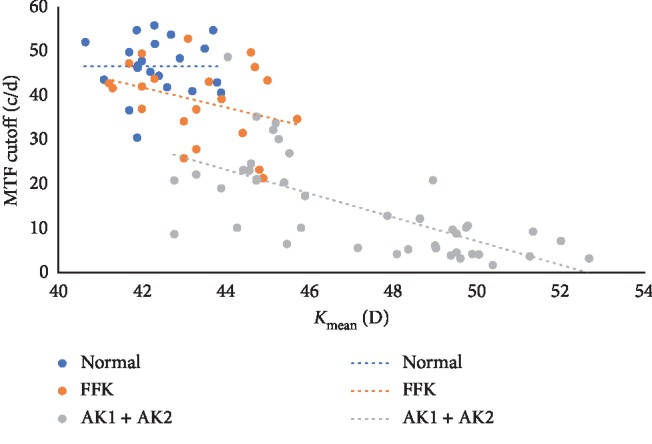
Correlation between the MTF cutoff and *K*_mean_ among normal, FFK, and keratoconus eyes (AK1 and AK2). A graph showing a significant correlation between the MTF cutoff and *K*_mean_ in keratoconus eyes (*r* = −0.710, *P* < 0.001). No significant correlation was found between the MTF cutoff and *K*_mean_ in the normal group (*r* = 0.004, *P*=0.987) and FFK group (*r* = −0.335, *P*=0.138).

Figure 1 showed the MTF cutoff was closely correlated to *K*_mean_ in keratoconus eyes (AK1 and AK2) (*r* = −0.710, *P* < 0.05), while the MTF cutoff showed no significant correlation to *K*_mean_ in the normal group (*r* = 0.004, *P*=0.987) and FFK group (*r* = −0.335, *P*=0.138). The relationships between other optical quality parameters and *K*_mean_ are shown in Supplemental [Supplementary-material supplementary-material-1].

### 3.5. ROC Curve Analyses

**Figure 2:**
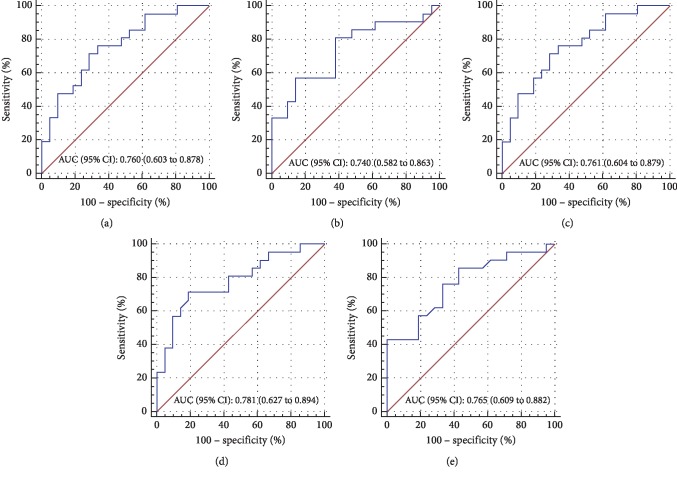
Comparisons between the normal group and FFK. Receiver operating characteristic (ROC) curves were constructed by plotting sensitivity versus 100% specificity at varying cutoff values of the MTF cutoff (a), SR (b), OV-100 (c), OV-20 (d), and OV-9 (e), respectively. Area under the curve (AUC) and 95% CI are noted at the bottom right of each graph. For further information about cutoff values, sensitivity, specificity, and Youden index, see Supplemental [Supplementary-material supplementary-material-1].

**Figure 3:**
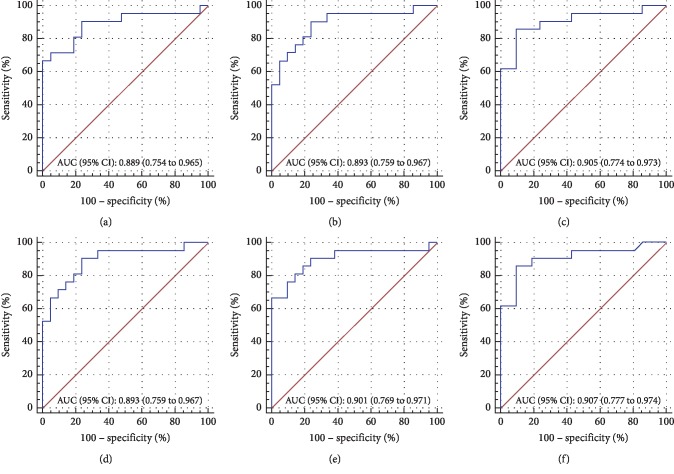
Comparisons between FFK and AK1. Receiver operating characteristic (ROC) curves were constructed by plotting sensitivity versus 100% specificity at varying cutoff values of the OSI (a), MTF cut off (b), SR (c), and OV-100 (d), OV-20 (e), OV-9 (f), respectively. Area under the curve (AUC) and 95% CI are noted at the bottom right of each graph. For further information about cut-off values, sensitivity, specificity, and Youden index, see Supplemental [Supplementary-material supplementary-material-1].

**Figure 4:**
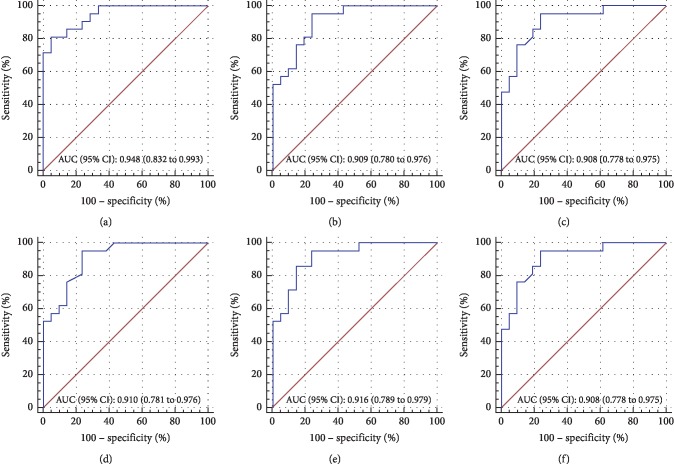
Comparisons between AK1 and AK2. Receiver operating characteristic (ROC) curves were constructed by plotting sensitivity versus 100% specificity at varying cutoff values of the OSI (a), MTF cutoff (b), SR (c), OV-100 (d), OV-20 (e), and OV-9 (f), respectively. Area under the curve (AUC) and 95% CI are noted at the bottom right of each graph. For further information about cutoff values, sensitivity, specificity, and Youden index, see Supplemental [Supplementary-material supplementary-material-1].

Then, we sought to evaluate the optical quality parameters as adjunctive diagnostic indicators by using ROC analysis (Figures 2–4). Comparing the MTF cutoff, SR, OV-100, OV-20, and OV-9 in normal eyes to FFK, the areas under the curve (AUC) were 0.760, 0.740, 0.761, 0.781, and 0.765, respectively (all *P* < 0.001) (Figure 2). Moreover, the OSI, MTF cutoff, SR, OV-100, OV-20, and OV-9 showed a significant ability to discern AK1 from FFK with the AUC of 0.889, 0.893, 0.905, 0.893, 0.901, and 0.907, respectively (all *P* < 0.001) (Figure 3). In addition, the OSI, MTF cutoff, SR, OV-100, OV-20, and OV-9 also demonstrated a significant ability to discern AK2 from AK1 with the AUC of 0.948, 0.909, 0.908, 0.910, 0.916, and 0.908, respectively (all *P* < 0.001) (Figure 4). The cutoff value, sensitivity, specificity, and Youden index of these parameters are shown in Supplemental Tables [Supplementary-material supplementary-material-1]–[Supplementary-material supplementary-material-1], respectively.

## 4. Discussion

Our findings showed that OQAS-II had good repeatability in measuring visual quality of normal, FFK, AK1, and AK2. The visual quality in FFK, AK1, and AK2 was inferior to that in normal. Our results showed that the MTF cutoff was significantly associated with *K*_mean_ in keratoconus eyes. In addition, our results demonstrated that the OQAS-II might help clinicians to better understand the visual quality in keratoconus and could be a useful tool for detecting FFK and monitoring its progression.

Keratoconus is a progressive corneal ectasia characterized by localized corneal thinning which leads to the protrusion of cornea [2]. Corneal tomography is currently the most widely available method to diagnose early keratoconus. Although the changes on the topography of cornea could be obviously detected before the clinical signs of keratoconus, these changes do not correlate with the visual acuity [18]. As far as we know, the wavefront sensors and OQAS-II were both objective evaluating devices of visual quality. While in eyes where scattered light and aberrations are prominent, wavefront sensors may overestimate image quality [19]. In contrast, the OQAS-II can reflect a more accurate description of the visual quality and has been widely used for clinical application [20–22]. OQAS-II images contained all the information about the visual quality of the eye including all the higher-order aberrations and scattered light, being both generally missed by most aberrometric techniques. In addition, the OQAS-II can also help clinicians explain why some patients have good BCVA, but the subjective visual disturbance is obvious. Therefore, it has been considered as a convenient and objective method for visual quality assessment.

In order to assess the visual quality and explore the potential diagnostic value of OQAS-II in keratoconus patients, the measurement repeatability of OQAS-II needs to be explored to make a reliable clinical judgement. Several studies have identified a good measurement repeatability of OQAS-II. Xu et al. [23] measured 119 healthy eyes with OQAS-II and concluded that the OQAS-II showed excellent repeatability for objective measurements of overall visual quality in clinic. Iijima et al. [24] also reported a good repeatability of OQAS in healthy adults. Furthermore, studies have also shown that the measurement repeatability of the DP system was good in FFK [12], which was in accordance with our current findings. To our knowledge, there are no studies on the measurement repeatability of OQAS-II in mild and moderate keratoconus. In clinic, ICC ≥ 0.75 indicates good to excellent repeatability, and ICC ≥ 0.90 means the device has excellent repeatability [25]. Our study showed that the repeatability of all the optical quality parameters detected by OQAS-II was excellent in AK1, while some parameters including MTF cutoff, SR, OV-100, OV-20, and OV-9 in AK2 showed good measurement repeatability. This might be attributed to the obvious irregular corneal distortion in AK2, consequently degrading the retinal image quality [26]. Our findings support that OQAS-II measurements are reliable in evaluating the visual quality changes in eyes with FFK, mild, and moderate keratoconus. Further studies on different stages of keratoconus should be performed to confirm our findings.

Then, we comparatively evaluated the visual quality in FFK, mild, and moderate keratoconus patients. The significant upward trend in OSI and downward trend in MTF cutoff, SR, OV-100, OV-20, and OV-9 observed from normal to moderate keratoconus indicated that as the disease continued to advance, the visual quality in keratoconus declined gradually. Our results showed that the MTF cutoff, SR, OV-100, OV-20, and OV-9 in FFK were significantly lower than those in the normal group (all *P* < 0.05). These results were consistent with those given by Ye et al. [12], but they did not evaluate the OSI value. Leonard et al. [13] compared the OSI values between the keratoconus eyes and normal eyes and found statistically significant increments of OSI scores in the AK1 and AK2 group, which was also consistent with our results. However, our results further showed the OSI had no significant difference between the FFK and normal group. Miháltz et al. [27]also reported that visual quality in terms of the Strehl ratio and the spot radius was degraded in the subclinical keratoconus and keratoconus group compared with that in the control group; although related, the Strehl ratio parameter described in their study was different from our results. Moreover, our results showed that the MTF cutoff was significantly associated with *K*_mean_ in keratoconus eyes. Further studies with different populations should be conducted to confirm the findings.

The ROC curve analysis could illustrate the diagnostic ability of a binary classifier system [28]. To evaluate the potential diagnostic value of OQAS-II parameters in FFK, mild, and moderate keratoconus, we made the ROC curve analyses of these parameters in our study. And we found some optical quality parameters except OSI displayed a significant ability to discern FFK from the normal group, and all the optical quality parameters displayed a significant ability to discern AK1 from FFK and AK2 from AK1, which indicated that the optical quality parameters could help to evaluate the progression of keratoconus and detect the early stage of the disease. The OV-20 had the largest area under the curve (AUC = 0.781) in FFK compared to the normal group, followed by the OV-9, while the OV-9 had the largest area under the curve (AUC = 0.907) in identifying AK1 from FFK. The OSI had the largest area under the curve (AUC = 0.948) in identifying AK2 from AK1. Regardless of any correlation between keratoconus age and stage, the significant upward trend in OSI and downward trend in MTF cutoff, SR, OV-100, OV-20, and OV-9 observed from normal to AK2 and significant AUC by ROC analysis (Figures 2–4) indicated that the optical quality parameters measured by OQAS-II may be helpful to monitoring the progression of keratoconus.

Several limitations of our study need to be addressed. Firstly, the sample size of the present study was small, which might affect the validity of our results. Secondly, we did not include the severe keratoconus eyes in our study since it is difficult to measure eyes with high astigmatism, which might get some bias of the visual quality through an external cylindrical lens. Thirdly, the mean OSI value recorded over 19.5 seconds without blinking was not included in current study, which reflects the tear film dynamic alterations and might be different among normal, FFK, mild, and moderate keratoconus. A further multicenter study should be conducted later.

In conclusion, the repeatability of OQAS-II was good in normal as well as FFK, mild, and moderate keratoconus eyes. And the FFK, mild, and moderate keratoconus patients had worse visual quality compared with that in normal eyes. Furthermore, the OQAS-II might be a new method in detecting FFK and a useful tool for objectively evaluating the progression of keratoconus.

## Figures and Tables

**Table 1:** Demographic data of subjects.

	NL (*n* = 21)	FFK (*n* = 21)	AK1 (*n* = 21)	AK2 (*n* = 21)	*P*					
					FFK/NL	AK1/NL	AK2/NL	FFK/AK1	FFK/AK2	AK1/AK2
Gender (male, %)^a^	71.43	80.95	80.95	71.43	0.525	0.525	1	1	0.525	0.525
Age (mean ± SD, years)^b^	22.67 ± 4.18	20.43 ± 4.88	21.95 ± 2.96	21.19 ± 4.97	0.098	0.594	0.272	0.257	0.570	0.570
Spherical (mean ± SD, D)^b^	−3.74 ± 1.64	−4.29 ± 2.41	−4.48 ± 2.26	−6.39 ± 4.92	0.576	0.449	0.007^*∗*^	0.852	0.035^*∗*^	0.051
Cylindrical (mean ± SD, D)^b^	−0.34 ± 0.40	−0.75 ± 0.35	−2.66 ± 2.21	−4.55 ± 1.71	0.405	0.000^*∗*^	0.000^*∗*^	0.000^*∗*^	0.000^*∗*^	0.000^*∗*^
*K*_s_ (mean ± SD, D)^b^	42.88 ± 0.94	43.86 ± 1.49	45.94 ± 1.25	51.97 ± 1.60	0.020^*∗*^	0.000^*∗*^	0.000^*∗*^	0.000^*∗*^	0.000^*∗*^	0.000^*∗*^
*K*_f_ (mean ± SD, D)^b^	41.96 ± 0.86	42.80 ± 1.24	43.57 ± 1.09	47.51 ± 1.67	0.034^*∗*^	0.000^*∗*^	0.000^*∗*^	0.049^*∗*^	0.000^*∗*^	0.000^*∗*^
*K*_mean_ (mean ± SD, D)^b^	42.39 ± 0.87	43.43 ± 1.34	44.75 ± 1.04	49.73 ± 1.24	0.010^*∗*^	0.000^*∗*^	0.000^*∗*^	0.000^*∗*^	0.000^*∗*^	0.000^*∗*^
TCT (mean ± SD, mm)^b^	544.10 ± 41.26	507.76 ± 28.09	468.29 ± 36.12	432.29 ± 20.77	0.001^*∗*^	0.000^*∗*^	0.000^*∗*^	0.000^*∗*^	0.000^*∗*^	0.001^*∗*^
IOP (mean ± SD, mmHg)^b^	14.22 ± 2.36	13.42 ± 1.88	12.52 ± 2.53	10.78 ± 2.68	0.293	0.024^*∗*^	0.000^*∗*^	0.240	0.001^*∗*^	0.021^*∗*^
Axial length (mean ± SD, mm)^b^	25.51 ± 0.87	25.53 ± 1.13	25.31 ± 0.72	24.92 ± 1.19	0.942	0.517	0.057	0.487	0.057	0.204
BCVA (logMAR) (mean ± SD)^b^	0.00 ± 0.00	0.02 ± 0.05	0.18 ± 0.24	0.50 ± 0.28	0.790	0.002^*∗*^	0.000^*∗*^	0.005^*∗*^	0.000^*∗*^	0.000^*∗*^

NL: normal; FFK: forme fruste keratoconus; AK1: stage 1 of the Amsler-Krumeich scales; AK2: stage 2 of the Amsler-Krumeich scales; *K*_s_: steep keratometry; *K*_f_: flat keratometry; *K*_mean_: mean central keratometry readings; TCT: thinnest corneal thickness; IOP: intraocular pressure; BCVA: best-corrected visual acuity; logMAR: logarithm of the minimal angle of resolution; ^a^chi-square test; ^b^one-way analysis of variance with LSD corrections. ^*∗*^*P* < 0.05 denotes statistical significance between the two groups.

**Table 2:** Measurement of repeatability of OQAS-II parameters for the normal group, FFK, AK1, and AK2.

	NL			FFK			AK1			AK2		
	ICC	RC	CV_w_ (%)	ICC	RC	CV_w_ (%)	ICC	RC	CV_w_ (%)	ICC	RC	CV_w_ (%)
OSI	0.973 (0.944 to 0.988)	0.152	12.448	0.968 (0.935 to 0.986)	0.303	18.053	0.994 (0.988 to 0.997)	0.971	12.306	0.934 (0.864 to 0.971)	4.740	17.319
MTF cutoff (c/d)	0.965 (0.929 to 0.985)	5.472	4.231	0.915 (0.824 to 0.963)	12.289	11.432	0.953 (0.903 to 0.979)	10.879	17.877	0.792 (0.570 to 0.909)	9.840	49.131
SR	0.868 (0.726 to 0.942)	0.088	11.712	0.887 (0.766 to 0.951)	0.088	14.011	0.950 (0.897 to 0.978)	0.088	24.325	0.770 (0.525 to 0.900)	0.088	52.705
OV-100	0.966 (0.930 to 0.985)	0.175	4.054	0.915 (0.823 to 0.963)	0.411	11.471	0.953 (0.904 to 0.980)	0.361	17.861	0.791 (0.568 to 0.909)	0.328	49.301
OV-20	0.957 (0.912 to 0.981)	0.232	6.915	0.910 (0.813 to 0.961)	0.361	13.725	0.963 (0.921 to 0.984)	0.248	18.254	0.779 (0.543 to 0.903)	0.232	52.291
OV-9	0.928 (0.851 to 0.969)	0.215	10.328	0.900 (0.794 to 0.956)	0.248	15.227	0.947 (0.891 to 0.977)	0.175	21.809	0.785 (0.555 to 0.906)	0.152	54.772

NL: normal; FFK: forme fruste keratoconus; AK1: stage 1 of the Amsler-Krumeich scales; AK2: stage 2 of the Amsler-Krumeich scales; ICC: intraclass correlation coefficient; RC: Repeatability; CV_w_: coefficient of variation; OSI: object scatter index; MTF cutoff: modulation transfer function cutoff; SR: Strehl ratio; OV-100: OQAS values at contrasts of 100%; OV-20: OQAS values at contrasts of 20%; OV-9: OQAS values at contrasts of 9%.

**Table 3:** OQAS-II parameters among the normal, FFK, AK1, and AK2 group.

	NL (*n* = 21)	FFK (*n* = 21)	AK1 (*n* = 21)	AK2 (*n* = 21)	*P*					
					FFK/NL	AK1/NL	AK2/NL	FFK/AK1	FFK/AK2	AK1/AK2
OSI	0.44 ± 0.18	0.61 ± 0.37	2.85 ± 2.62	9.88 ± 3.86	0.815	0.001^*∗*^	0.000^*∗*^	0.003^*∗*^	0.000^*∗*^	0.000^*∗*^
MTF cutoff (c/d)	46.69 ± 6.43	38.81 ± 8.93	21.97 ± 10.59	7.23 ± 4.51	0.002^*∗*^	0.000^*∗*^	0.000^*∗*^	0.000^*∗*^	0.000^*∗*^	0.000^*∗*^
SR	0.27 ± 0.05	0.23 ± 0.06	0.13 ± 0.05	0.06 ± 0.02	0.002^*∗*^	0.000^*∗*^	0.000^*∗*^	0.000^*∗*^	0.000^*∗*^	0.000^*∗*^
OV-100	1.56 ± 0.21	1.29 ± 0.30	0.73 ± 0.35	0.24 ± 0.15	0.002^*∗*^	0.000^*∗*^	0.000^*∗*^	0.000^*∗*^	0.000^*∗*^	0.000^*∗*^
OV-20	1.21 ± 0.22	0.95 ± 0.26	0.49 ± 0.27	0.16 ± 0.10	0.000^*∗*^	0.000^*∗*^	0.000^*∗*^	0.000^*∗*^	0.000^*∗*^	0.000^*∗*^
OV-9	0.75 ± 0.16	0.59 ± 0.17	0.29 ± 0.16	0.10 ± 0.07	0.000^*∗*^	0.000^*∗*^	0.000^*∗*^	0.000^*∗*^	0.000^*∗*^	0.000^*∗*^

NL: normal; FFK: forme fruste keratoconus; AK1: stage 1 of the Amsler-Krumeich scales; AK2: stage 2 of the Amsler-Krumeich scales; OSI: object scatter index; MTF cutoff: modulation transfer function cut off; SR: Strehl ratio; OV-100: OQAS values at contrasts of 100%; OV-20: OQAS values at contrasts of 20%; OV-9: OQAS values at contrasts of 9%. ^*∗*^*P* < 0.05 denotes statistical significance.

## Data Availability

The data used to support the findings of this study are available from the corresponding author upon request.

## Supplementary Materials

Supplementary Materials Supplemental Table 1: Amsler-Krumeich clasification for keratoconus severity. Supplemental Table 2: relationship between *K*_mean_ and OQAS-II parameters Supplemental Table 3: the ROC analysis of OQAS-II parameters between FFK and NL. Supplemental Table 4: the ROC analysis of OQAS-II parameters between FFK and AK1. Supplemental Table 5: the ROC analysis of OQAS-II parameters between AK1and AK2.
