# Supplementary material for: Assessment of Visual Quality in Eyes with Forme Fruste Keratoconus and Mild and Moderate Keratoconus Based on Optical Quality Analysis System II Parameters
Source: J Ophthalmol. 2020 Feb 29;2020:7505016. doi: 10.1155/2020/7505016 (PMC7125474; doi:10.1155/2020/7505016)
Supplement: Supplementary Materials — Supplemental Table 1: Amsler-Krumeich clasification for keratoconus severity. Supplemental Table 2: relationship between Kmean and OQAS-II parameters Supplemental Table 3: the ROC analysis of OQAS-II parameters between FFK and NL. Supplemental Table 4: the ROC analysis of OQAS-II parameters between FFK and AK1. Supplemental Table 5: the ROC analysis of OQAS-II parameters between AK1and AK2. [file 7505016.f1.docx]

**Supplemental Table 1.** **Amsler-Krumeich Clasification for keratoconus Severity^a^**

| **Stage** | **Findings** |
| --- | --- |
| 1 | Eccentric steepening |
|  | Myopia, induced astigmatism, or both < 5.00D |
|  | Mean central K readings < 48.00D |
| 2 | Myopia, induced astigmatism, or both from 5.00D to 8.00D |
|  | Mean central K readings <53.00D |
|  | Absence of scarring |
|  | Corneal thickness > 400 microns |
| 3 | Myopia, induced astigmatism, or both from 8.00 to 10.00 D |
|  | Mean central K readings > 53.00D |
|  | Absence of scarring |
|  | Corneal thickness 300 to 400 microns |
| 4 | Refraction not measurable |
|  | Mean central K readings > 55.00D |
|  | Central corneal scarring |
|  | Corneal thickness < 200 microns |

D: diopters; K: keratometry; ^a^Criteria used for grading keratoconus severity^15^.

**Supplemental Table 2. Relationship between Kmean and OQAS-II parameters**

| **Kmean** | | | | | | |
| --- | --- | --- | --- | --- | --- | --- |
| **Parameters** | **NL** | | **FFK** | | **Keratoconus** | |
|  | *r* | *P* | *r* | *P* | *r* | *P* |
| OSI | -0.073 | 0.753 | 0.426 | 0.054 | 0.759 | 0.000* |
| MTF cut off (c/d) | 0.004 | 0.987 | -0.335 | 0.138 | -0.710 | 0.000* |
| SR | 0.029 | 0.900 | -0.468 | 0.032* | -0.702 | 0.000* |
| OV-100 | 0.005 | 0.982 | -0.335 | 0.138 | -0.713 | 0.000* |
| OV-20 | -0.002 | 0.994 | -0.436 | 0.048* | -0.715 | 0.000* |
| OV-9 | 0.011 | 0.962 | -0.509 | 0.019* | -0.708 | 0.000* |

NL: normal; FFK: forme fruste keratoconus; Keratoconus: AK1 and AK2; OSI: Object scatter index; MTF cut off: Modulation transfer function cut off; SR: Strehl ratio; OV-100: OQAS values at contrasts of 100%; OV-20: OQAS values at contrasts of 20%; OV-9: OQAS values at contrasts of 9%;

**P* < 0.05 denotes statistical significance

**Supplemental Table 3. The ROC analysis of OQAS-II parameters between FFK and NL.**

| **Parameters** | **Cut-off** | **Sensitivity(%)** | **Specificity(%)** | **Youden index** | **AUC(95%*CI*)** |
| --- | --- | --- | --- | --- | --- |
| MTF cut off (c/d) | 43.458 | 71.43 | 71.43 | 0.429 | 0.760(0.603 to 0.878) |
| SR | 0.265 | 80.95 | 61.90 | 0.429 | 0.740(0.582 to 0.863) |
| OV-100 | 1.45 | 71.43 | 71.43 | 0.429 | 0.761(0.604 to 0.879) |
| OV-20 | 1.05 | 71.43 | 80.95 | 0.524 | 0.781(0.627 to 0.894) |
| OV-9 | 0.53 | 42.86 | 100.00 | 0.429 | 0.765(0.609 to 0.882) |

FFK: forme fruste keratoconus; NL: normal; MTF cut off: Modulation transfer function cut off; SR: Strehl ratio; OV-100: OQAS values at contrasts of 100%; OV-20: OQAS values at contrasts of 20%; OV-9: OQAS values at contrasts of 9%; AUC: Area under the curve

**Supplemental Table 4. The ROC analysis of OQAS-II parameters between FFK and AK1.**

| **Parameters** | **Cut-off** | **Sensitivity(%)** | **Specificity(%)** | **Youden index** | **AUC(95%*CI*)** |
| --- | --- | --- | --- | --- | --- |
| OSI | 0.89 | 90.48 | 76.16 | 0.667 | 0.889(0.754 to 0.965) |
| MTF cut off (c/d) | 33.84 | 90.48 | 76.19 | 0.667 | 0.893(0.759 to 0.967) |
| SR | 0.151 | 85.57 | 90.48 | 0.761 | 0.905(0.774 to 0.973) |
| OV-100 | 1.130 | 90.48 | 76.19 | 0.667 | 0.893(0.759 to 0.967) |
| OV-20 | 0.500 | 66.67 | 100 | 0.667 | 0.901(0.769 to 0.971) |
| OV-9 | 0.377 | 85.71 | 90.48 | 0.762 | 0.907(0.777 to 0.974) |

FFK: forme fruste keratoconus; AK1: stage 1 of the Amsler-Krumeich scales; OSI: Object scatter index; MTF cut off: Modulation transfer function cut off; SR: Strehl ratio; OV-100: OQAS values at contrasts of 100%; OV-20: OQAS values at contrasts of 20%; OV-9: OQAS values at contrasts of 9%; AUC: Area under the curve

**Supplemental Table 5. The ROC analysis of OQAS-II parameters between AK1 and AK2.**

| **Parameters** | **Cut-off** | **Sensitivity(%)** | **Specificity(%)** | **Youden index J** | **AUC(95%*CI*)** |
| --- | --- | --- | --- | --- | --- |
| OSI | 8.262 | 80.95 | 95.24 | 0.762 | 0.948(0.832 to 0.993) |
| MTF cut off (c/d) | 12.863 | 95.24 | 76.19 | 0.714 | 0.909(0.780 to 0.976) |
| SR | 0.097 | 95.24 | 76.19 | 0.714 | 0.908(0.778 to 0.975) |
| OV-100 | 0.427 | 95.24 | 76.19 | 0.714 | 0.910(0.781 to 0.976) |
| OV-20 | 0.223 | 85.71 | 85.71 | 0.714 | 0.916(0.789 to 0.979) |
| OV-9 | 0.210 | 95.24 | 76.19 | 0.714 | 0.908(0.778 to 0.975) |

AK1: stage 1 of the Amsler-Krumeich scales; AK2: stage 2 of the Amsler-Krumeich scales; OSI: Object scatter index; MTF cut off: Modulation transfer function cut off; SR: Strehl ratio; OV-100: OQAS values at contrasts of 100%; OV-20: OQAS values at contrasts of 20%; OV-9: OQAS values at contrasts of 9%; AUC: Area under the curve
